# New Medical Journals

**Published:** 1860-11

**Authors:** 


NEW MEDICAL JOURNALS.—We have received the first two numbers of the *Columbus Review of Medicine and Surgery*, a bi-monthly journal of ninety-six pages, edited by Dr. W. L. McMillen. This is the second medical periodical existing in Columbus, and from the character of its contents we can safely predict that it will become one of the leading journals of the West. We congratulate the editor on the auspicious beginning of his enterprise, and trust that future numbers will sustain the good opinion we have formed of it.

The *London Medical Review* is the title of another monthly candidate for favor, the first number of which was issued in July by the enterprising and experienced publisher, Mr. Baillière. As is usual with foreign periodicals, the name of the editor does not appear on its cover, but from the names of the contributors to its pages, it is easy to perceive that it is under the management of able men. It gives us pleasure to place it on our exchange list.

